# Behavioral and psychosocial effects of rapid genetic counseling and testing in newly diagnosed breast cancer patients: Design of a multicenter randomized clinical trial

**DOI:** 10.1186/1471-2407-11-6

**Published:** 2011-01-10

**Authors:** Marijke R Wevers, Margreet GEM Ausems, Senno Verhoef, Eveline MA Bleiker, Daniela EE Hahn, Frans BL Hogervorst, Rob B van der Luijt, Heiddis B Valdimarsdottir, Richard van Hillegersberg, Emiel JTh Rutgers, Neil K Aaronson

**Affiliations:** 1 Division of Psychosocial Research and Epidemiology, The Netherlands Cancer Institute, PO Box 90203, 1006 BE Amsterdam, The Netherlands; 2 Division of Medical Genetics, University Medical Center Utrecht, PO Box 85090, 3508 AB Utrecht, The Netherlands; 3 Division of Diagnostic Oncology, The Netherlands Cancer Institute, PO Box 90203, 1006 BE Amsterdam, The Netherlands; 4 Division of Medical Oncology, The Netherlands Cancer Institute, PO Box 90203, 1006 BE Amsterdam, The Netherlands; 5 Oncological Sciences Department, Mount Sinai School of Medicine, Icahn Medical Institute, 1425 Madison Avenue, New York, NY 10029, USA; 6 Division of Surgery, University Medical Center Utrecht, PO Box 85500, 3508 GA Utrecht, The Netherlands; 7 Division of Surgical Oncology, The Netherlands Cancer Institute, PO Box 90203, 1006 BE Amsterdam, The Netherlands

## Abstract

**Background:**

It has been estimated that between 5% and 10% of women diagnosed with breast cancer have a hereditary form of the disease, primarily caused by a *BRCA1 *or *BRCA2 *gene mutation. Such women have an increased risk of developing a new primary breast and/or ovarian tumor, and may therefore opt for preventive surgery (e.g., bilateral mastectomy, oophorectomy). It is common practice to offer high-risk patients genetic counseling and DNA testing after their primary treatment, with genetic test results being available within 4-6 months. However, some non-commercial laboratories can currently generate test results within 3 to 6 weeks, and thus make it possible to provide *rapid *genetic counseling and testing (RGCT) prior to primary treatment. The aim of this study is to determine the effect of RGCT on treatment decisions and on psychosocial health.

**Methods/Design:**

In this randomized controlled trial, 255 newly diagnosed breast cancer patients with at least a 10% risk of carrying a *BRCA *gene mutation are being recruited from 12 hospitals in the Netherlands. Participants are randomized in a 2:1 ratio to either a RGCT intervention group (the offer of RGCT directly following diagnosis with tests results available before surgical treatment) or to a usual care control group. The primary behavioral outcome is the uptake of direct bilateral mastectomy or delayed prophylactic contralateral mastectomy. Psychosocial outcomes include cancer risk perception, cancer-related worry and distress, health-related quality of life, decisional satisfaction and the perceived need for and use of additional decisional counseling and psychosocial support. Data are collected via medical chart audits and self-report questionnaires administered prior to randomization, and at 6 month and at 12 month follow-up.

**Discussion:**

This trial will provide essential information on the impact of RGCT on the choice of primary surgical treatment among women with breast cancer with an increased risk of hereditary cancer. This study will also provide data on the psychosocial consequences of RGCT and of risk-reducing behavior.

**Trial registration:**

The study is registered at the Netherlands Trial Register (NTR1493) and ClinicalTrials.gov (NCT00783822).

## Background

### Breast cancer and genetics

Breast cancer is the most frequently occurring malignancy in women in the Netherlands. Approximately 1 in 8 women will develop the disease during their lifetime, resulting in approximately 12,000 new cases annually. A positive family history of breast cancer is the single strongest risk factor for developing the disease. In 5%-10% of the cases, breast cancer has a hereditary basis. In 15%-30% of patients from high-risk families, breast cancer is caused by a germline mutation in the *BRCA1 *or *BRCA2 *gene [1-4]. A similar percentage of breast cancers is expected to have a genetic basis due to mutations in (combinations of) other low penetrance breast cancer susceptibility genes [5]. Current estimates indicate that women who carry mutations in the *BRCA1 *or *BRCA2 *genes have up to an 85% lifetime risk of developing breast cancer, and can also have up to a 60% lifetime risk of developing ovarian cancer [6,7]. Breast cancer patients who carry a *BRCA1/2 *mutation have a 3% annual risk of developing contralateral breast cancer, with the overall risk being as high as 52% by the age of 70 [7,8]. For *BRCA1/2 *carriers who were diagnosed with breast cancer before the age of 40 years, the cumulative risk of developing contralateral breast cancer after a follow up of 25 years is even higher, i.e. 63% [9]. Given these high rates of new primary cancers, breast cancer patients with strong family histories of the disease may benefit from genetic counseling and testing as a means of making a well-informed decision on preventive measures.

### Genetic counseling and testing in breast cancer patients

The objectives of cancer genetic counseling, in general, are to improve knowledge and understanding of the possible genetic basis of the disease, of personal risks of developing cancer, and of the possible consequences of undergoing genetic testing [10,11]. In the case of breast cancer, the goal of genetic counseling is to ensure that women have been sufficiently educated regarding inherited breast/ovarian cancer to make informed decisions concerning genetic testing, and available preventive and treatment options [12,13]. The majority of published studies have found that genetic counseling is effective in increasing knowledge and awareness of cancer risk, and of the consequences of genetic testing [11,14-16]. Factors that may be indicative of hereditary breast cancer are young age at diagnosis, multiple family members with breast cancer, male relatives with breast cancer, breast and ovarian cancer in the family and/or breast cancer and prostate cancer in family members at a relatively young age. Women with one or more of these factors are considered to have a 10% risk or greater of carrying a *BRCA1 *or *BRCA2 *gene mutation, and are thus, in general, considered as candidates for DNA testing. Genetic counseling and DNA testing for breast cancer usually takes approximately 4-6 months to complete. However, some hospitals and non-commercial laboratories are now able to generate test results within 3 to 6 weeks. This technology of *rapid *genetic testing creates new opportunities for providing both women and their treating surgeons with information potentially relevant for deciding between available treatment options, including type of surgery and adjuvant therapy [17-19].

Newly diagnosed patients who learn that they are gene mutation carriers may decide to undergo a mastectomy rather than a lumpectomy, or bilateral mastectomy (BLM) rather than unilateral mastectomy. Recent studies have shown that BLM and delayed contralateral prophylactic mastectomy (CPM) substantially reduce the risk of contralateral breast cancer, while studies on survival have yielded mixed results [20-23]. Additionally, women who choose unilateral or bilateral mastectomy as their initial surgical treatment will often be spared the necessity of undergoing radiation therapy. However, rapid genetic counseling and testing (RGCT) may necessitate some delay in surgical treatment until DNA test results are available. Previous studies have indicated that the majority of newly diagnosed breast cancer patients at high risk of being *BRCA1/2 *mutation carriers will accept the invitation to undergo RGCT, and that the DNA test results can have a substantial impact on the choice of surgery [3,24,25]. In these British and American studies, between one-half and two-thirds of the breast cancer patients carrying a *BRCA1 *or *BRCA2 *mutation opted for a bilateral mastectomy, with some opting for a delayed CPM instead of direct BLM. Additionally, between 16% and 24% of high-risk patients who test negative for a *BRCA1/2 *mutation opted for BLM [3,25].

The psychosocial impact of genetic testing in recently diagnosed breast cancer patients has been the subject of only limited study [26]. Although disclosure of test results may increase short term psychological distress, there is no evidence to suggest that there is a sustained increase in levels of distress as a result of such counseling or testing [26-28]. A Dutch study showed that an active approach to genetic counseling in patients recently diagnosed with breast cancer and in an early stage of primary treatment (i.e. 7-8 weeks after surgery), did not increase psychological stress significantly [27,28]. In addition, in a small pilot study in 8 women who were offered RGCT following their breast cancer diagnosis but prior to surgery, none reported that the offer of RGCT had added significantly to the stress that they were already experiencing due to the diagnosis of breast cancer [29]. There is also relatively little known about the psychological impact of BLM or CPM. The few studies performed, to date, all but one of which was retrospective in nature, have reported high levels of satisfaction with CPM and no adverse effects on health-related quality of life (HRQL) [30-32].

To summarize, recent advances in the technology of genetic testing for breast cancer open the possibility of offering women with a suspected hereditary form of the disease the opportunity to undergo RGCT in the period of time between receiving the diagnosis and undergoing primary treatment. For those high-risk women who opt for direct BLM or delayed CPM, there are clear advantages in terms of reduction in the risk of contralateral disease, although an improvement in survival has not yet been convincingly demonstrated [20,22]. Women who choose for (bilateral) mastectomy can also avoid having to undergo radiotherapy. This, in turn, can improve the results of breast reconstruction [33]. However, there are insufficient data available on the impact of RGCT, when offered routinely to high-risk women, on medical decision-making, and there is relatively little known about the impact of RGCT on cancer-specific distress, treatment satisfaction, and HRQL.

### Objectives and research hypotheses

This study is evaluating the behavioral and psychosocial effects of the routine offer of RGCT to newly diagnosed primary breast cancer patients who meet criteria for being at-risk of having a *BRCA1 *or *BRCA2 *gene mutation. RGCT is being offered to women shortly after diagnosis, prior to surgery. The comparison group is composed of women who receive usual care (UC), in which RGCT is available but is rarely recommended or requested.

The specific research hypotheses are:

1. A significantly greater percentage of women in the RGCT group as compared to the UC group will undergo a direct bilateral mastectomy (BLM).

2. A significantly greater percentage of women in the RGCT group as compared to the UC group will undergo a delayed prophylactic contralateral mastectomy (CPM).

3. Women in the RGCT group will report significantly lower levels of perceived cancer risk, cancer worries, and cancer-related distress at 12 month follow-up than women in the UC group.

4. Women in the RGCT will have significantly higher levels of knowledge of genetic issues in breast cancer, and higher levels of decisional satisfaction than women in the UC group.

5. Following from hypotheses 1 and 2, women in the RGCT group will report significantly more problems with body image and sexuality than those in the UC group. No significant differences will be observed between the RGCT and the UC groups on other HRQL outcomes.

## Methods/Design

This is a multicenter, randomized, controlled trial in which patients are randomized to RGCT or to UC on a 2:1 basis. The primary clinical outcomes include: the uptake of direct BLM or of delayed CPM. Data on surgical outcomes will be abstracted from the medical records. This protocol follows the CONSORT guidelines [34].

**Figure 1:**
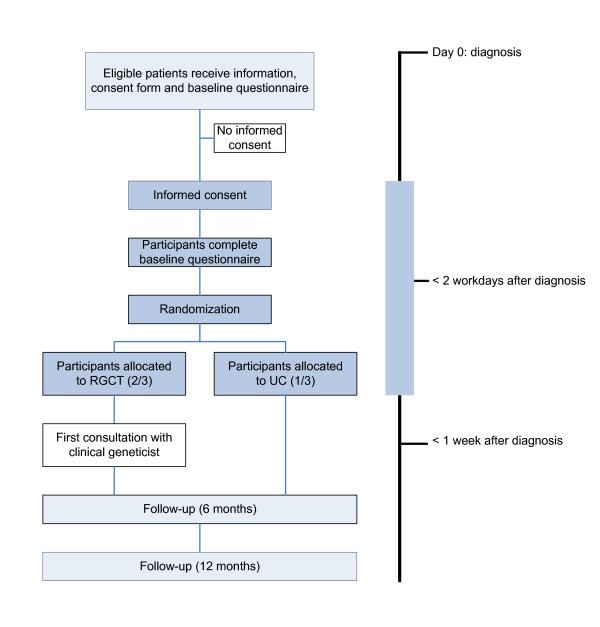
**Study flow diagram**.

Psychosocial outcomes include: perceived risk for ipsilateral and contralateral breast cancer and for ovarian cancer, cancer-related worry and distress; knowledge of genetic aspects of breast cancer; decisional satisfaction; and HRQL. The study also is evaluating women's experience of and satisfaction with RGCT (i.e., the timing and quality of the services provided, the perceived impact on treatment decisions, the perceived need for additional psychosocial support, etc.), and the surgeons' experience with RGCT. Standardized questionnaires are administered at study entry (prior to randomization), and at 6 month and 12 month follow-up (see figure 1). The institutional review boards of all participating hospitals have approved the study. The study is registered at the Netherlands Trial Register (NTR1493) and ClinicalTrials.gov (NCT00783822).

### Participants

The study sample will be composed of 255 women with a clinically confirmed, newly diagnosed primary breast cancer or ductal carcinoma in situ (DCIS) who meet at least one of the criteria for referral for genetic counseling and DNA analysis according to the Dutch Institute for Healthcare Improvement (CBO). These criteria are: (1) age at time of breast cancer diagnosis less than 35 years; (2) previous history of cancer in the contralateral breast, with the first breast cancer diagnosis at less than 50 years of age; (3) previous personal history of ovarian cancer; (4) a family history of ovarian cancer; (5) a first degree male relative with breast cancer; (6) breast cancer diagnosed under the age of 50 years, with a first degree relative diagnosed with breast cancer under the age of 50 years; (7) breast cancer diagnosed under the age of 50 years, with a relative with prostate cancer diagnosed under the age of 60 years; (8) breast cancer, with two or more relatives from the same side of the family diagnosed with breast cancer, where at least one family member (either the patient or a relative) was diagnosed under the age of 50 years; or (9) a proven *BRCA1/2 *mutation in the family. Women who meet one or more of these criteria are considered to have a 10% or greater chance of having a BRCA1/2 gene mutation [35,36]. Women are excluded from the study if they are younger than 18 years of age, lack basic fluency in the Dutch language, are incapable of understanding informed consent, or are diagnosed with a breast cancer recurrence or with metastastic disease. The patients are being recruited from 12 hospitals in the Amsterdam and Utrecht regions of the Netherlands.

### Recruitment and randomization

Patient recruitment began in November 2008, following approval by the ethical review committees of the participating hospitals. Participating surgeons make use of an 11-item checklist to determine patient eligibility. All patients who meet the study inclusion criteria are informed by the surgeon or nurse practitioner about the study, and are given an envelope containing an information letter, a consent form and the baseline questionnaire. Eligible patients receive a phone call by a member of the research team within a few days, in order to carry out the informed consent procedures and to administer the baseline questionnaire to consenting patients. Patients are then randomized to the RGCT or the UC group in a 2:1 ratio. The computerized randomization is stratified per hospital and blocked per 9 cases [37]. These 12 random allocation sequences are generated by an independent individual, are stored in computer files, and remain unknown to the researchers until the patient is randomized. The participant as well as the treating surgeon or nurse practitioner is informed about the randomization outcome. The researchers are blinded to randomization outcome at the time of the baseline assessments, but not at the follow-up assessments. This is due to the fact that the researchers are responsible for arranging appointments with the genetic counselor for those women assigned to the RGCT arm of the study.

### Intervention

Women in the RGCT group receive an appointment with a clinical geneticist or genetic counselor within one week following randomization, either at the Family Cancer Clinic of the Netherlands Cancer Institute/Antoni van Leeuwenhoek Hospital or at the Department of Medical Genetics of the University Medical Centre Utrecht. Those patients who decline to undergo RGCT are given the opportunity to receive genetic counseling and testing at a later time.

While the standard steps involved in genetic counseling are followed, the process is accelerated when necessary. All patients are asked to complete a standard, detailed family history form in order to draw a three-generation pedigree. Where possible, confirmation of the (age at) diagnosis of the affected family members is confirmed via medical record audits following required informed consent procedures. At the time of the first consultation with the clinical geneticist, the urgency of the steps to be taken are discussed and a decision about DNA testing is made. If the patient agrees, a blood sample for testing for mutations in the *BRCA1 *and *BRCA2 *genes can be taken on the same day. For women whose test result will not be of immediate consequence for their choice of treatment, the timing of the DNA testing does not need to be accelerated (i.e., DNA test results will be made available within 4 months). Examples are women who, from a medical point of view, cannot opt for breast conserving therapy and have already had a contralateral mastectomy, women who do not wish to take immediate secondary preventive measures, or women whose family history provides them with sufficient reasons to undergo bilateral treatment regardless of whether a mutation can be found.

Where it is anticipated that the DNA test results could influence treatment decisions, a fast-track procedure is used and the results are made available within 3 to 6 weeks. From a medical point of view, this timeframe is sufficiently short to inform patients and clinicians about the results before definite treatment decisions have to be made.

The number of contacts between the patient and the clinical geneticist prior to surgery depends on the complexity of each individual case. For women who choose not to undergo DNA testing, this may be limited to a single session. For women who undergo DNA testing, at least one additional session is held in order to discuss the DNA test results. All results of the RGCT are forwarded to the treating surgeon, and consultation between the clinical geneticist and surgeon takes place prior to and following the patient's first appointment with the clinical geneticist.

Psychosocial support is offered routinely to all women who opt for rapid DNA testing, *BRCA1/2 *carriers and to all women who are considering undergoing BLM regardless of whether they have undergone DNA testing. Where appropriate, referrals are made to mental health caregivers in the hospital. The need for and uptake of more intensive psychosocial care during subsequent treatment are being assessed as part of the study.

Women in the UC condition receive standard advice and care from their treating surgeon. In relation to the possible hereditary nature of their disease, this typically involves discussion of the family history and of appropriate surgical options. In some cases, patients may be referred for genetic counseling or may self-refer. However, in current practice, this occurs rarely during the pre-surgery period [38,39].

### Study measures

#### Sociodemographic and clinical data

The patients' age, education, marital status, ethnic background, work status and general health status are obtained via the baseline questionnaire. Data on family pedigree and genetic risk status are obtained via a questionnaire. Clinical data, including diagnosis, tumor characteristics and treatment history (surgical treatment, surgical complications, breast reconstruction, radiotherapy, adjuvant chemotherapy or hormonal therapy) are obtained via both questionnaire and medical record audit. Results of the genetic counseling and testing, including genetic test results, are obtained from the records of the participating departments of clinical genetics.

#### Choice of surgical procedure

The patients' definitive surgical treatment decision is determined via medical record audit. Via questionnaires women are asked to report the factors motivating their choice of treatment.

*Risk perception *is assessed with 4 items adapted from previous studies [40-42] that inquire about current perceived risk of recurrent or contralateral breast cancer (all questionnaires), of ovarian cancer, and of the possibility of having hereditary breast cancer (follow-up questionnaires). Women are asked to rate on a 5-point scale how likely they think it is that they will develop cancer again, and to rate their perceived risk on a continuous scale from 0% to 100%.

*Cancer worries *are assessed with 8 items, 6 of which are adapted from previous work by Watson [40,43], that measure the frequency of cancer-related worries, their impact on mood, and their impact on daily functioning.

The Hospital Anxiety and Depression Scale (HADS) [44] is used to assess *psychological distress. *The HADS contains 14 items. Both a total score and separate scores for anxiety and depression can be calculated. The HADS has been validated for use in the Dutch population, showing good psychometric properties, with Cronbach's alphas for both the anxiety and depression scales >0.70 [45-47].

*Cancer-specific distress *is assessed with the Impact of Events Scale (IES), a 15-item Likert-scale organized into 2 subscales: intrusive thoughts and feelings, and avoidance of thoughts and feelings related to the stressful situation. In this case, the stressful situation is the diagnosis of breast cancer [48]. The IES has high levels of internal consistency (alpha's above 0.80) and has been translated and validated for use in the Dutch setting [49].

*Satisfaction with decision-making *is assessed with the Satisfaction with Decision Scale (SWD) [50] and the Decisional Conflict Scale [51,52]. The 6-item SWD-scale can be adapted to any medical decision and, in this case, refers to the choice of surgical treatment. The scale has high internal consistency reliability (alpha = 0.86) [50]. The 16-item Decisional Conflict Scale contains three subscales: 'Uncertainty', 'Factors contributing' and 'Effective decision making'. The scale had high internal consistency reliability (alpha of the three subscales ranges from 0.75-0.82 in the Dutch version).

*Knowledge of breast cancer and its genetic aspects *is assessed with a 7-item, true-false-don't know questionnaire adapted from Claes *et al. *[14,53].

*Health-related quality of life *is assessed with the EORTC QLQ-C30 [54] and the EORTC breast cancer module, the QLQ-BR23 [55]. The QLQ-C30 includes 5 functional scales, 3 symptom scales, and a number of single item symptom measures. It is designed for use with a broad range of cancer patient populations. The QLQ-BR23 assesses breast cancer-specific symptoms, treatment side-effects, body image, sexuality and future perspective. Both questionnaires have been used extensively in studies of women with breast cancer, and exhibit good levels of reliability (alpha coefficients between 0.70 and 0.85) and construct validity.

#### Process-related variables

Perceived influence of the surgeon on treatment choice is assessed with a series of questions adapted from Schwartz et al. [24]. Women in the RGCT arm of the study are asked a series of study-specific questions about their experience and satisfaction with RGCT, and their perceived need for additional psychosocial care. Psychosocial care actually received is also being determined from the medical records and via self-report. The content of the professional psychosocial support received is also being assessed.

### Sample size

The sample size calculations are based primarily on the type of surgery chosen, and specifically on the uptake of bilateral mastectomy. With a total sample of 255 women (170 in the RGCT arm and 85 in the UC arm), the study will have 80% power to detect a difference of 18% versus 5% in the uptake of bilateral mastectomy, with the p-value set at 0.05. The sample size ratio of 2:1 was chosen to ensure that the RGCT sample is sufficiently large to obtain stable estimates of bilateral mastectomy and delayed prophylactic contralateral mastectomy uptake among *BRCA1/2 *carriers, and to allow for secondary analyses (e.g., to examine predictors of DNA testing uptake). The percentages indicated above are based on the following assumptions: (1) based on the participating hospitals' experiences in the previous three years, 5% of the women in the UC arm are expected to choose bilateral mastectomy; (2) in the RGCT arm, it is expected that 20% will test positive for a *BRCA1 *or *BRCA2 *mutation, of whom, on average, 50% will opt for a bilateral mastectomy. Of the remaining 80% of women in the RGCT arm, we expect a 10% uptake of bilateral mastectomy. For the remaining, primarily self-report outcomes (e.g., cancer worries and cancer-specific distress, decisional satisfaction, HRQL), the sample of 255 participants is more than sufficient to detect a 0.5 standard deviation between-group difference in mean scores. Based on data obtained from the cancer registries of the Integral Cancer Centers of Amsterdam and Middle Netherlands, and on data provided by the participating hospitals, it is estimated that there will be a total of 2550 newly diagnosed breast cancer patients during the recruitment period. Approximately 15% of these women is expected to be eligible for participation. Based on prior experience with recruitment of patients into similar studies, we expect an 80% response rate. This results in an expected N of 306 patients. This allows for an attrition rate of 17%, while retaining 255 women for the primary analysis.

### Statistical analyses

Analyses will first be performed to evaluate the comparability of the RGCT and the UC groups at study entry in terms of sociodemographic and clinical characteristics. Student's t-test or appropriate non-parametric statistics will be used, depending on the level of measurement. If the groups are not comparable on one or more background variables, those variables will be employed routinely as covariates in subsequent analyses. Between group differences in choice of surgery will be tested using the chi-square statistic or, if statistical adjustment is required for covariates, multiple logistic regression analysis. Analysis of (co)variance will be employed to assess group differences at 6 and 12 month follow-up in risk perception, psychological distress, knowledge, decisional satisfaction, and HRQL. Where appropriate, sociodemographic and clinical background variables, as well as baseline scores on the outcomes of interest, will be used as covariates. Effect sizes will be calculated using standard statistical procedures. Supplementary, descriptive analyses (both quantitative and qualitative) will be used for reporting the patients' experiences with RGCT. All primary analyses will, to as great an extent as possible, be conducted on an intention-to-treat basis. That is, all women in the RGCT group will be included in that group in the analyses, regardless of whether they actually carried through with RGCT. Supplementary analyses will be undertaken to examine sociodemographic, clinical and psychosocial factors associated significantly with (non-) attendance at the RGCT and uptake of BLM.

## Discussion

It is common practice to refer breast cancer patients with suspected hereditary breast cancer for genetic counseling and testing after their initial treatment has been completed [38]. Women found to have a mutation in the *BRCA1 *or *BRCA2 *gene may opt for additional preventive surgery following their initial surgical treatment. A complicating factor in delaying genetic counseling and testing until after the completion of primary treatment is that, when that treatment includes radiotherapy, additional, preventive surgical procedures (e.g., mastectomy with breast reconstruction) may be quite challenging for the plastic surgeon [33]. By offering genetic counseling and testing in the period between breast cancer diagnosis and initial surgical treatment, it is possible to incorporate the results of those procedures into treatment decision-making. It is important to note that genetic counseling, whether done prior to or following primary treatment, is non-directive in nature. The intent is to provide women with information about the possible hereditary basis of their disease, so that they can make informed choices regarding primary surgical and adjuvant treatment, as well as possible preventive surgery. However, little information is currently available on the benefits and potential problems associated with RGCT from the patients' perspective. It is important to determine these effects now, as it is anticipated that RGCT will be increasingly common place, as non-commercial genetic labs gear up to be able to generate DNA test results in only a matter of weeks. Moreover, rapid genetic counseling, in and of itself, may influence treatment decisions. It should be noted that the results of RGCT are not only expected to influence the choice of surgical treatment, but may also have an impact on chemotherapy options as well. Specific chemotherapeutic agents such as PARP-inhibitors are being developed that have antitumor activity in *BRCA*-associated cancer [56].

### Methodological considerations

The major methodological strength of this study is the use a randomized controlled trial design which will allow a rigorous test of the effects of RGCT with optimal internal validity. The relatively large sample size and the use of primarily standardized measures represent additional strengths of the study. Finally, the trial's multicenter nature should increase the external validity or generalizability of the study findings.

A limitation of this study is the relatively short period of follow-up (12 months). While this is sufficient for examining the effect of RGCT on primary treatment choice and on short term psychosocial outcomes, it does not permit longer term follow-up on these latter outcomes.

## Conclusion

This study is expected to provide important information on the impact of RGCT among newly diagnosed breast cancer patients on the choice of surgical treatment and on psychosocial well-being. Since RGCT is anticipated to become common practice in the future, the results of this study can contribute to improving the quality of multidisciplinary breast cancer care.

## Competing interests

NutsOhra Foundation, a charitable fund linked to the private insurance company NutsOhra, has financially supported this study. There are no other competing interests.

## Authors' contributions

NKA, MGEMA and SV are the principal investigators of this study. MRW is the doctoral student on this study, and generated the first draft of this manuscript based on the study protocol. EMAB, DEEH, and HBV contributed to the development of the study protocol. EJThR and RvH are the lead surgeons. FH and RBvdL are the collaborating molecular geneticists.

All authors approved the final version of the manuscript.

## Pre-publication history

The pre-publication history for this paper can be accessed here:

http://www.biomedcentral.com/1471-2407/11/6/prepub
